# Oklahoma

**Published:** 1896-09

**Authors:** 


*Oklahoma.* The Board of Regents of the Agricultural College, the Governor, and the Territorial Veterinarians have decided that Texas fever exists in Oklahoma, and have ordered the Kiowa, Comanche, Wichita, Ponca, Otoe, Kaw, and Osage Indian reservations and the Counties of Cleveland, Pottawatomie, Canadian, Oklahoma, Lincoln, Payne, Logan, Noble, and Pawnee quarantined, and have prohibited the taking of cattle from these sections into other parts of the Territory under heavy penalty.

